# Aggressive Presentation of Streptococcus constellatus

**DOI:** 10.7759/cureus.14534

**Published:** 2021-04-17

**Authors:** Abhinav K Vulisha, Riya Sam, Hassan Nur, Neharika Bhardwaj, Srija Sirineni

**Affiliations:** 1 Internal Medicine, Advocate Illinois Masonic Medical Center, Chicago, USA; 2 Internal Medicine, Bhaskara Medical College, Hyderabad, IND

**Keywords:** streptococcus constellatus, deep vein thrombosis (dvt), pulmonary decortication, prolonged antibiotics

## Abstract

The *Streptococcus anginosus* group (SAG) consists of three bacteria (*Streptococcus intermedius*, *Streptococcus constellatus*, and *Streptococcus anginosus*) that are known commensals of the upper respiratory, digestive, and reproductive tracts. While a rare occurrence, these bacteria have the capability of causing devastating pyogenic infections and ensuing abscess formations. It is often difficult to distinguish this group as a contaminant or the offending organism (as it is often cultured in respiratory specimens); therefore, it is important to understand the risk factors, clinical presentation, and diagnostic findings that can provide a more accurate picture to identify the organism. Published literature pertaining to the SAG group has rarely documented any invasive surgical intervention that was undertaken for treatment. We describe a case of a 59-year-old male who presented for persistent chest pain and profuse productive cough weeks after he was diagnosed with a left lower extremity deep vein thrombosis and right-sided pulmonary embolism. The patient was found to have a rapidly evolving *Streptococcus constellatus* right middle lobe lung abscess complicated by a right hemithorax empyema. Management included an exploration of the right chest, decortication, parietal pleurectomy, and partial excision of the right middle lobe. Subsequently, the patient completed four weeks of antibiotics with ertapenem.

## Introduction

*Streptococcus anginosus* group (SAG) is a group of gram-positive streptococci that normally colonizes the upper respiratory, digestive and, reproductive tracts [1]. The group consists of three different species: *S. anginosus*, *S. constellatus*, and *S. intermedius* [1]. The most important clinical feature of these micro-organisms is their tendency to cause suppurative infections at various sites, ranging from dental to deep visceral abscesses particularly involving the lungs [1]. SAG typically affects the lungs through aspiration of oral secretions, direct implantation by trauma or surgery, extension by continuity, or hematogenous dissemination [2]. Underlying risk factors such as periodontal disease, diabetes mellitus, neoplasms, alcohol abuse, HIV, and chronic obstructive pulmonary disease (COPD) reportedly predispose patients to SAG lung infections [2]. In our case, it is unclear whether the recent pulmonary embolism (PE) with infarction was the primary risk factor for the *S. constellatus* lung infection.

## Case presentation

**Figure 1:**
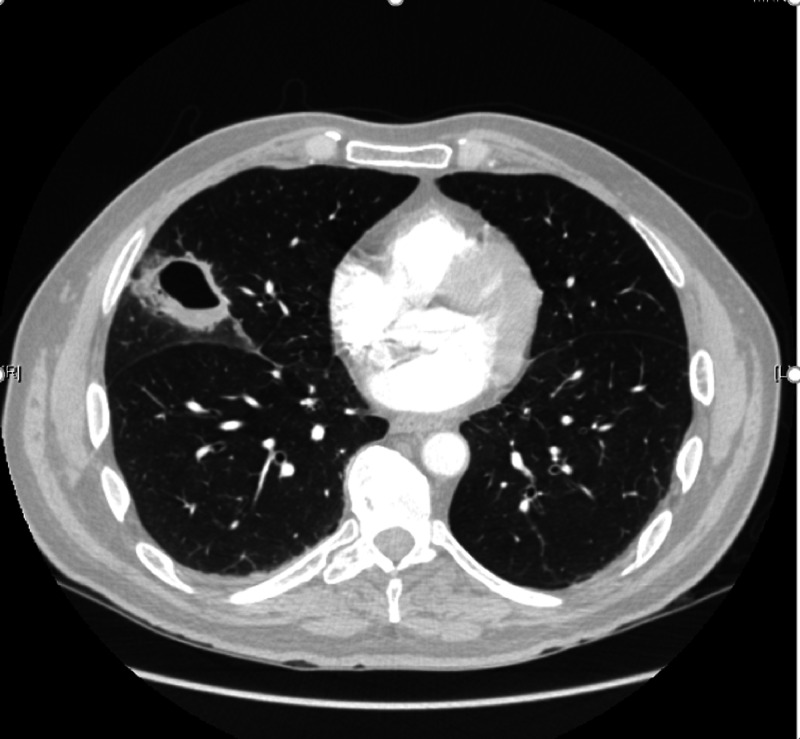
CT of the chest showing a 4.0 x 4.6 cm cavitating lesion in the right middle lobe with associated, surrounding ground-glass opacities in the transverse plane.

**Figure 2:**
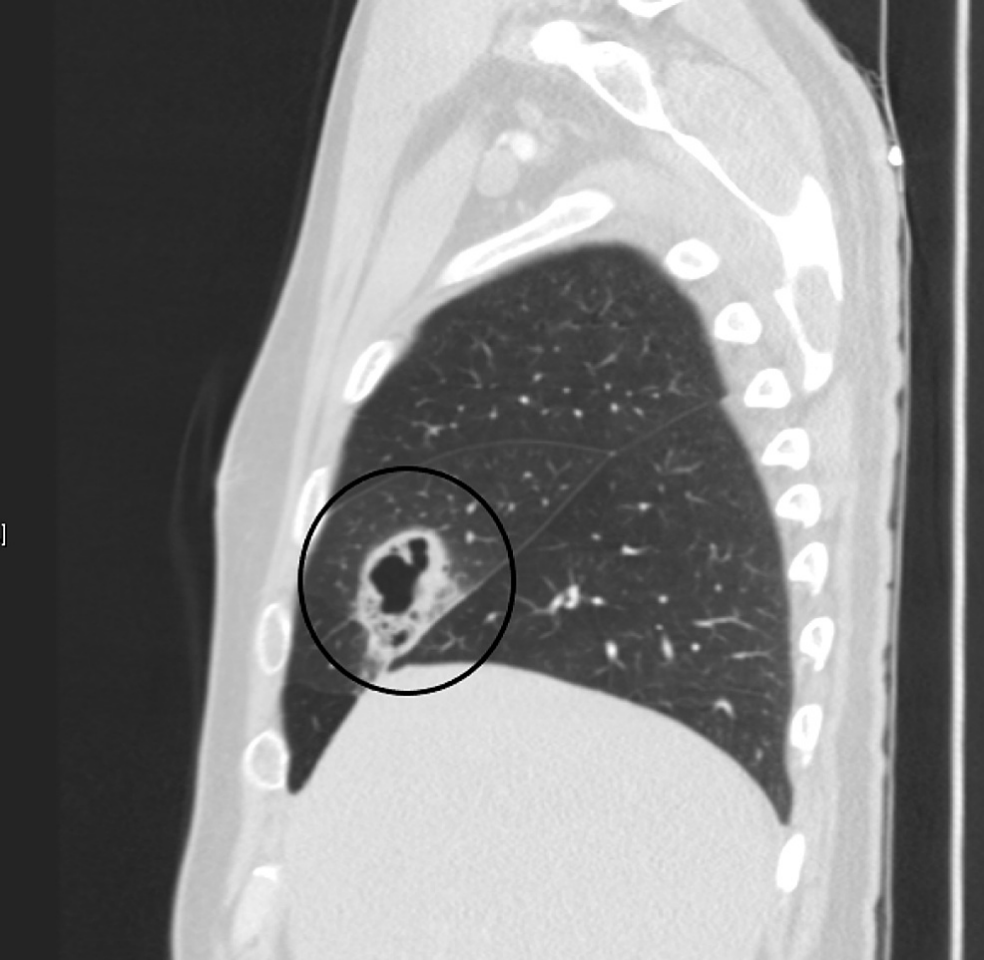
CT of the chest showing a 4.0 x 4.6 cm cavitating lesion in the right middle lobe with associated, surrounding ground-glass opacities in the coronal plane.

A 57-year-old male with a past medical history of hepatitis C and 30 pack-year smoking history (quit one month prior to ED visit) presented to the emergency department with left lower extremity pain/swelling and was found to have an acute deep vein thrombosis extending from the left superficial femoral vein to the left popliteal vein with involvement of the left deep calf veins. During the hospital course, a chest CTA (computed tomography angiography) with contrast was obtained, which demonstrated acute pulmonary emboli in the right main pulmonary artery extending into the segmental branches. The patient was started on Eliquis® and discharged with instruction to follow up with the hematology department. He subsequently presented with worsening productive cough and pleuritic chest pain three weeks later. Vitals were stable, and laboratory investigation were essentially unremarkable including a normal white blood cell (WBC) and procalcitonin. The CTA of the chest demonstrated a new 4.6 cavitating lesion in the right middle lobe (Figures 1, 2), and the patient was discharged on the same day with advice to follow up in one week. Ten days later, the patient presented again to the ED with persistent cough associated initially with hemoptysis, which later progressed to brownish green sputum. The patient quantified the volume of sputum as one-third of an Arizona tea bottle. He also admitted to unintentional weight loss of 20 lbs in the interim.

On admission, vitals were within normal limits aside from sinus tachycardia (heart rate: 100s). His breath sounds were diminished on the right middle to lower lung fields. Rest of the physical examination was unremarkable. Laboratory investigation included hemoglobin of 12.8 g/dL (reference range: 13-17 g/dL), WBC count of 18 K/mcL (reference range: 4.2-11 K/mcL), and procalcitonin of 3.5 ng/mL (reference range: ≤ 0.10 ng/mL).

**Figure 3:**
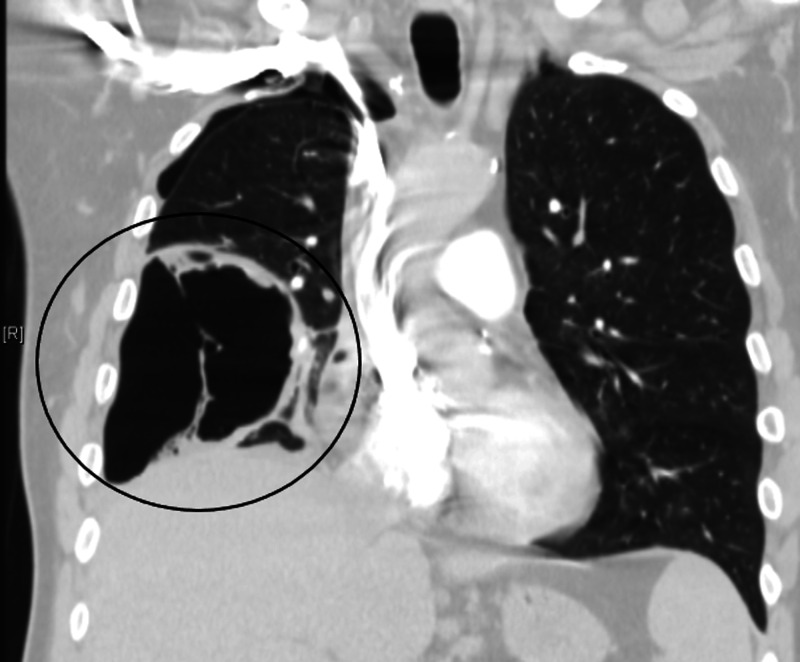
CT of the chest showing significant interval worsening of cavitary right middle lobe lesion, now demonstrating an internal air-fluid levels and gas-filled septations and measuring up to 18.6 cm. This likely represents evolution of lung necrosis with probable superimposed infection.

**Figure 4:**
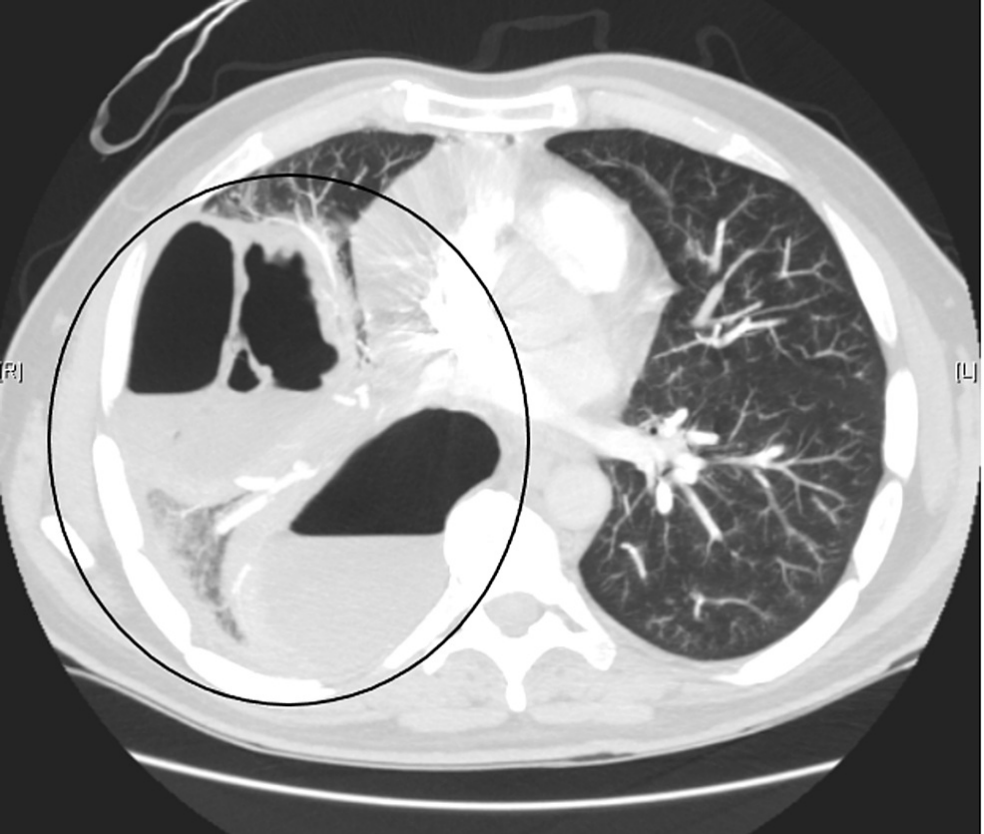
CT of the chest showing significant interval worsening of cavitary right middle lobe lesion, now demonstrating an internal air-fluid levels and gas-filled septations and measuring up to 18.6 cm. This likely represents evolution of lung necrosis with probable superimposed infection. New small hydropneumothorax along the convexity of the right upper lung is also noted.

Chest radiograph demonstrated a multi-loculated thick-walled cavitary lesion in the right middle lobe and a new air collection with a large air-fluid level in the posterior medial right lower lobe. CTA with contrast revealed significant interval worsening and increased size of the right middle lobe cavitary lesion, which had now extended into the right lower lobe and showed internal air-fluid levels and gas-filled septations, measuring up to 18.6 cm (Figures 3, 4).

**Figure 5:**
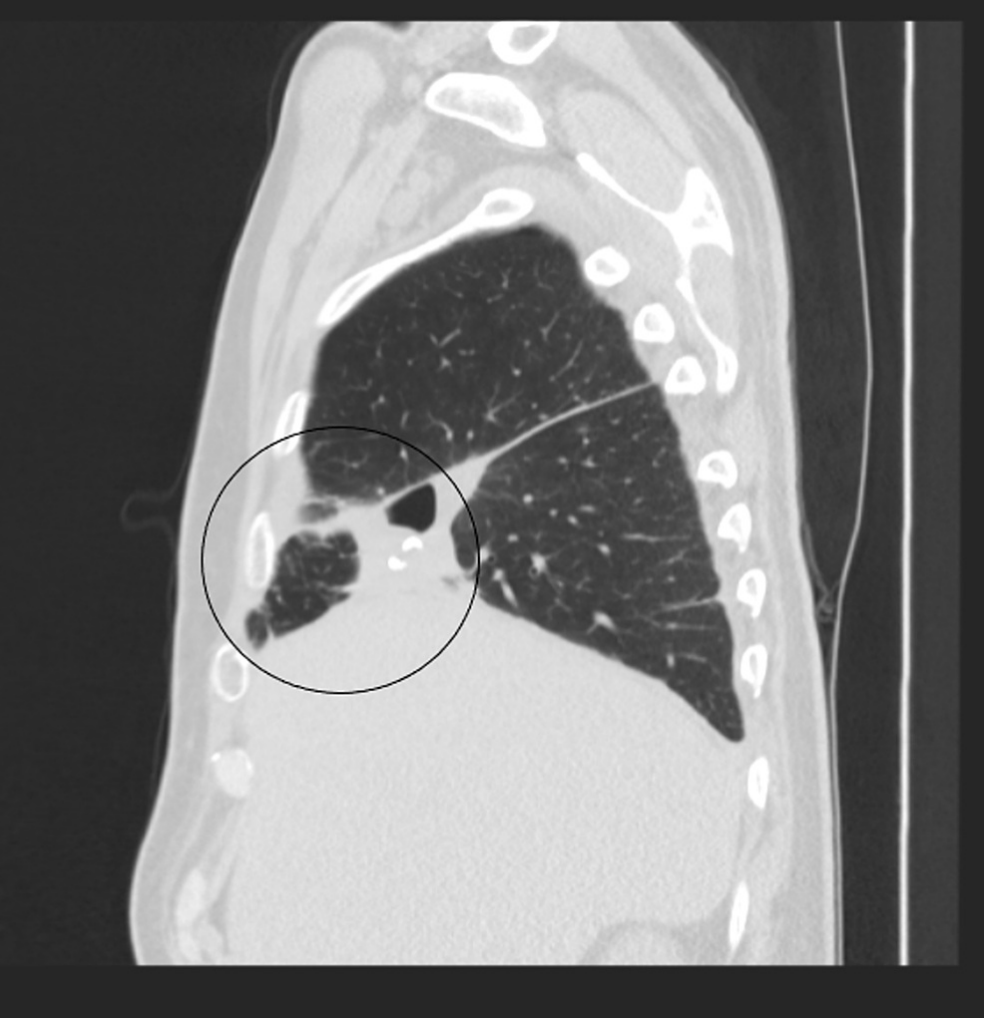
CT of the chest one month later shows a small residual collection of air and possible fluid in the right lateral lower lung near the major fissure in the region of the previous large abscess.

The patient was started on piperacillin-tazobactam and vancomycin, with the latter being discontinued given low suspicion, negative MRSA (methicillin-resistant *Staphylococcus aureus*), and blood cultures with no growth at five days. The patient continued to expectorate copious amounts of foul-smelling sputum during the course of hospitalization. Lab investigations into common lung pathologies such community-acquired pneumonias, common viral pathogens, and tuberculosis, including four samples of acid-fast bacilli (AFB), QuantiFERON, and mycobacterial culture, were largely negative. Initially, a heparin drip was initiated for therapeutic anticoagulation, and, thereafter, the patient underwent placement of a retrievable inferior vena cava filter to cover the post-operative period (off anticoagulation). Surgical management was undertaken, which included an exploration of right hemi-chest, decortication, parietal pleurectomy, and partial excision of the right middle lobe given operative findings of right hemithorax empyema and liquefied middle lobe. Intraoperative cultures grew pan-sensitive *S. constellatus*. Pathological examination of pleura revealed pleural and adipose tissue demonstrating acute inflammation, necrosis, granulation, and organizing fibrinopurulent exudate. Ultimately, the patient was discharged on intravenous ertapenem to complete a 35-day course and was followed up by cardiothoracic surgery and infectious disease services for two months after completing the course (Figure 5).

## Discussion

*Streptococcus constellatus* is a member of group *Streptococcus milleri *group, a group of gram-positive cocci, which have a potential to cause invasive infection from the normally sterile site [3]. SAG mostly causes respiratory infections and are often difficult to distinguish from contaminants as they are often grown in respiratory specimens [1]. Although Kobo et al. [4] have published a short report on the pyogenic potential of different SAGs and found that *S. constellatus* was associated with bacteremia with no abscess or empyema formation, there have been a few reports of *S. constellatus* forming abscess/empyema.

Commonly identified risk factors/conditions predisposing to infection by *S. constellatus* include aspiration (most common), direct implantation from trauma, surgery, extension by contiguity, and hematogenous spread [2], wherein virulence is increased in the presence of anaerobes [5]. There have been case reports of* S. constellatus* causing liver abscess, cerebellar abscess [6,7], and empyema (most common diagnosis in 78%) caused by the *S. milleri* group [2]. Risk factors for such infections include male gender, history of smoking, alcohol abuse, neoplastic disease, chronic pulmonary disease (such as COPD), periodontal disease, diabetes mellitus, hepatitis, and HIV. Risk factors in our patient include a history of hepatitis C, smoking, and a recent diagnosis of PE.

Culture is the most common means of detecting bacteria. Multiplex PCR (polymerase chain reaction) has been used by Hatrongjit et al. [8] for simultaneous rapid detection of six clinically relevant* Streptococcal *species (*Streptococcus pneumoniae*, *Streptococcus suis*, *Streptococcus gallolyticus subsp. gallolyticus*, *S. gallolyticus subsp. pasteurianus*, *Streptococcus intermedius*, and *Streptococcus anginosus/constellatus*). The process takes three hours to identify an organism and is considered a simple, reliable, and efficient method for the detection of these six streptococci. In our patient, none of the blood or sputum cultures was positive, and the organism was identified only on cultures from tissue obtained during biopsy.

Clinical features of severe pneumonia were observed in our patient, which included high-grade fevers, cough with expectoration/hemoptysis, and loss of appetite. Additional features of weight loss, such as diaphoresis, were also observed. Routine investigations to rule out differentials such as *Mycobacterium tuberculosis*, influenza, respiratory pathogen panel of common viruses, and community-acquired pneumonias were negative. Combination treatment modalities with antibiotics and surgery are required in most of the patients. Thoracotomy was needed in 75% of patients in one of the studies [2]. Choice and duration of antibiotics have varied in different case reports. Most patients required a long-term duration of antibiotics, with the longest being nine months with commonly used antibiotics such as penicillin/B-lactam, carbapenem, clindamycin (most effective in severe periodontal infection) [9], ceftriaxone, metronidazole, and ciprofloxacin. Our patient was treated for 35 days with ertapenem after an initial course of penicillin/B-lactam. Highest resistance was observed to clindamycin in one of the retrospective studies conducted on orofacial infections [10].

## Conclusions

*Streptococcus constellatus*, a normal pathogen found in human body, has a potential to cause empyema, and necrosis of different organs such as the lungs, liver, and brain, especially when associated with comorbid conditions and risk factors. We highlight the unique presentation of our case due to multiple reasons. Firstly, our patient had very few risk factors and was considered immunocompetent. Secondly, the rapid progression of lesion despite being on broad-spectrum antibiotics was a particularly unique finding as the lesion grew nearly 10 cm within a short time frame and involved most of the right middle lobe. Finally, our patient had operative findings of right hemithorax empyema and middle lobe necrosis, which required extensive surgical intervention. The possibility of superimposed infection from aspiration in an underlying PE is the possible etiology in our patient. Further research and more data on the correlation between pulmonary emboli predisposing to *S. constellatus* lung infections would ascertain the risk and treatment in an efficient manner in such a patient population. When suspected, we recommend immediate surgical approach along with IV antibiotics in the management of similar presentation of* S. constellatus*.
